# Application of Solution NMR to Structural Studies on α-Helical Integral Membrane Proteins

**DOI:** 10.3390/molecules22081347

**Published:** 2017-08-15

**Authors:** Dae-Won Sim, Zhenwei Lu, Hyung-Sik Won, Seu-Na Lee, Min-Duk Seo, Bong-Jin Lee, Ji-Hun Kim

**Affiliations:** 1 Department of Biotechnology, College of Biomedical and Health Science, Konkuk University, Chungbuk 27478, Korea; konapapa@nate.com (D.-W.S.), wonhs@kku.ac.kr (H.-S.W.), leesn1029@naver.com (S.-N.L.); 2 Department of Biochemistry, Vanderbilt University, Nashville, TN 37204, USA; zhenwei.lu@vanderbilt.edu; 3 Department of Molecular Science and Technology & College of Pharmacy, Ajou University, Suwon 16499, Korea; mdseo@ajou.ac.kr; 4 The Research Institute of Pharmaceutical Sciences, College of Pharmacy, Seoul National University, Gwanak-gu, Seoul 151-742, Korea; 5 College of Pharmacy, Chungbuk National University, Cheongju 28160, Chungbuk, Korea

**Keywords:** solution NMR, α-helical integral membrane protein, structure, membrane-mimetics, isotope labeling

## Abstract

A large portion of proteins in living organisms are membrane proteins which play critical roles in the biology of the cell, from maintenance of the biological membrane integrity to communication of cells with their surroundings. To understand their mechanism of action, structural information is essential. Nevertheless, structure determination of transmembrane proteins is still a challenging area, even though recently the number of deposited structures of membrane proteins in the PDB has rapidly increased thanks to the efforts using X-ray crystallography, electron microscopy, and solid and solution nuclear magnetic resonance (NMR) technology. Among these technologies, solution NMR is a powerful tool for studying protein-protein, protein-ligand interactions and protein dynamics at a wide range of time scales as well as structure determination of membrane proteins. This review provides general and useful guideline for membrane protein sample preparation and the choice of membrane-mimetic media, which are the key step for successful structural analysis. Furthermore, this review provides an opportunity to look at recent applications of solution NMR to structural studies on α-helical membrane proteins through some success stories.

## 1. Introduction

Membrane proteins (MPs) represent ~30% of all proteins in living organisms [1]. They play many important roles in a wide range of cellular functions such as transport, transmission of cell signaling and scaffold support [2]. Due to their significant participation in physiological processes, MPs constitute ~60% of drug targets and are the most attractive object of research in the pharmaceutical industry [3]. Therefore, knowledge about the structure of MPs is decidedly important for efficient drug design. These structures have been mostly determined by X-ray crystallography approaches where impressive developments in the structural studies of MPs were achieved recently [4,5,6,7]. Other techniques such as electron microscopy and solution and solid state NMR have also contributed to determination of the structure of a few interesting MPs [8,9,10,11,12,13,14,15]. Although the number of MP structures deposited in the Protein Data Bank (PDB) has grown exponentially since the first MP structure was solved, these structures still represent less than 2% of the total number of structures; this state of affairs means that determination of MP structure remains a challenge at present. In this situation, NMR can be a powerful tool complementary to X-ray crystallography and electron microscopy (EM) for the determination of structures [16,17,18]. When compared with X-ray crystallography and EM, NMR can provide unique and interesting information such as protein dynamics on a wide range of time scales and interactions with internal or other components in the membrane-associated region, generally regarded as the “blind spot” in structural biology; these data offer a chance to understand the biological roles of MP [19]. All these information can be detected at the individual amino acid level (e.g., via recording intensity or chemical shift values) and are directly related to quality of the NMR spectra. Considering that crystallization processes of X-ray crystallography are basically based on the trial-and-error methods, the quality of NMR spectra can be a useful standard of judgement for obtaining high quality crystals. In the same context, these information can fill the gap between all-or-nothing results of crystallization screening. Meanwhile, when a low-resolution structure of protein complex composed of several proteins was obtained by EM spectroscopy, the quality of whole protein complex structure can be improved by fitting of high-resolution structure of unit protein determined by NMR spectroscopy. So far, many excellent reviews have addressed MP expression, NMR techniques, and the progress of structural studies using NMR [19,20,21,22,23,24]. This review will briefly provide guidelines on what needs to be done to determine the MP structure by solution NMR analysis, and discuss some details on membrane-mimetic media according to the analysis of the structures deposited in PDB.

## 2. General Approaches to Structural Studies on Integral MPs

**Figure 1:**
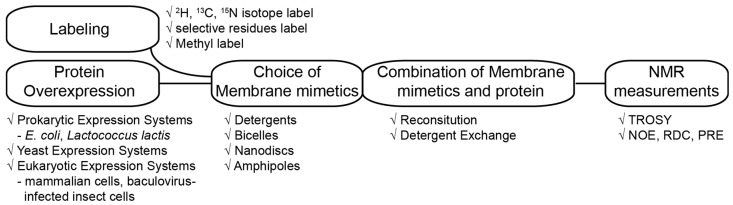
Flow chart for structure determination of MPs.

For the determination of integral MP structure using solution NMR, there are several steps that are important for the whole procedure: an overexpression system for the recombinant protein, a model membrane system, the method for sample purification, protein isotopes labelling, and a proper pulse program as described in Figure 1. To acquire results successfully, a sufficient amount of a stable functional recombinant protein should first be obtained. Nonetheless, structural studies using solution NMR methods generally require a relatively large amount of protein (~150 uL of concentration over 100 μM for the NMR sample in general) as compared to other methods. Moreover, eukaryotic MPs are frequently not overexpressed well or lose the function in an *Escherichia coli* (*E. coli*) expression system, which is the most commonly used one for structural studies. In this case, modification of the protein construct and/or switching to another expression system, such as yeast or eukaryotic system can be viable solutions [25,26,27]. In addition, minimal medium culture for the isotope labeling of protein possibly can also cause a reduction of target protein production. Unless, these problems are solved, it is impossible to move on to the next step. Although membrane protein expression is important, this topic will not be discussed any further in this review. Instead, several excellent reviews are directly available to the reader [20,22].

To determine tertiary structure of a protein, it is necessary to detect and assign NMR signals to proton, carbon and nitrogen atoms in amino acid residues. The naturally abundant atoms, ^12^C and ^14^N, are not suitable for protein NMR due to their spin quantum numbers, thus, amino acid residues of a protein should be labeled with ^13^C and/or ^15^N via Besides, specific labeling technologies have been employed to overcome the limit on size or to facilitate assignment of NMR signals.

Preparation of recombinant proteins should be followed by membrane mimetics screening. Suitable membrane mimetics provide a membrane environment in which the protein maintains its native structure as well as function and yields high-quality NMR spectra. By means of suitable pulse programs, better-quality NMR spectra and restraint information can be obtained for structure calculation. Transverse relaxation optimized spectroscopy (TROSY)-based pulse sequences combined with high-field NMR machine dramatically increase the membrane protein/detergent size limit of solution NMR for structural analysis [28] and can give a high-quality NMR spectra.

### 2.1. Protein Labeling

#### 2.1.1. Protein Deuteration

MPs behave like high molecular weight proteins owing to their binding to membrane mimetics, even though they are small in size. For this reason, serious peak broadening occurs frequently in the NMR spectra of MPs, which make these structural studies difficult. MP deuteration is widely used to improve the quality of NMR spectra. The biggest advantage of deuteration of a protein is a reduction in dipole-dipole relaxation, because the deuteron has a gyromagnetic ratio 6.5-fold lower than that of the proton [29,30]. In case of a carbon atom that is directly attached hydrogens, deuteron replacement of a proton reduces the strong dipole-dipole interactions of the attached proton, thus leading to a dramatic change in relaxation. For example, deuteration of a Cα carbon results in a tenfold decreases in the transverse relaxation rate for proteins [31]. Therefore, perdeuteration or even partial deuteration can facilitate multidimensional experiments involving extended coherence transfer schemes passing through aliphatic carbons, such as Cα in the CT-HNCA experiments and eventually results in a higher signal-to-noise because of a more rapid ^1^H transverse relaxation rate as well as improvement of ^13^C linewidths [29]. 

Deuteration of Cα up to an 80% level can be achieved by culturing cells in a D_2_O-containing medium, which is relatively inexpensive as compared to complete deuteration that requires the use of perdeuterated glucose in the expression medium. In practice, 80%-deuterated samples are often sufficient for experiments on an amide-detected backbone and side chain assignment experiments [32]. On the other hand, complete deuteration should be considered when experiments involve assignment of NMR signals to a side chain or methyl group using a pulse program that contains multiple transfer steps [32].

There are two additional things to consider when deuterating MPs. First, some crystallographic results suggest that deuteration of proteins can sometimes lead to changes in protein structure and function [33,34]. Second, a back-exchange of amide deuterons to protons should be used for detection at an amide proton site; however, well-packed transmembrane segments can make the D- > H back-exchange difficult, thus leading to disappearance of peaks [35,36].

#### 2.1.2. Methyl Labeling

The methyl labeling method—where perdueterated proteins are protonated only at amide positions, and at the methyl groups of Ile/Val/Leu residues, the proteins are subjected to ^13^C isotope labeling—was developed thanks to the efforts of Kay and colleagues [29,37]. This technology can be useful for studying the structure of MPs because methyl groups are located frequently in the hydrophobic core, which is intimately involved in the structure and dynamics of such proteins [38]. For research on the structural characteristics of MPs, methyl probes in particular have favorable properties: (1) concentration of each methyl group can be significantly increased; (2) methyl groups tend to be dynamics because these groups are located at the end of side chains, thereby allowing researchers to go beyond the limits of protein size in NMR studies [39,40]; (3) the technology of selective protonation of methyl groups against a highly deuterated background is well established; (4) methyl probes can help to determine distances between proximal groups that were seen far away in the primary structure [41,42]. Indeed, the development of labeling strategies has enabled solution NMR studies of supermolecular complexes up to 1 MDa. In practice, incorporation of a residue-specific methyl group into perdeuterated proteins can be achieved by means of specific precursors. For example, Ile-δ1 or Ile-γ2 methyl group can be achieved by means of 2-ketobutyrate or 2-hydroxy-2-ethyl-3-ketobutyrate, respectively. The details of methyl label strategies have been addressed in many excellent review [37,43]. On the other hand, some limitations of methyl labeling in α-helical MPs have been described [32]. First, assignment of methyl peaks in helical MPs is sometimes challenging. Second, most methyl-methyl nuclear Overhauser effect (NOE) cross-peaks lie near the diagonal and may be difficult to resolve in helical MPs. Third, an expensive perdeuterated detergent must be used for eliminating overlaps between aliphatic peaks of detergent and methyl peaks.

#### 2.1.3. Amino Acid Type Selective Labeling

The requisite assignments of NMR peaks in helical transmembrane proteins usually suffer from poor chemical shift dispersion and severe overlaps of peaks in the spectra. Amino acid-selective isotope labeling can aid in sequence-specific resonance assignments in crowd regions of NMR spectra. Several techniques have been developed to achieve highly efficient selective labeling, including cell-free protein synthesis [44], in vivo labelling using auxotrophic strains [45], and selective inhibition of amino acid synthesis [46]. Cell-free protein synthesis is certainly a useful method for producing cytotoxic proteins and offers a rather freely labeled pattern. Nevertheless, it also has drawbacks such as a low yield of protein production and expensive and labor-consuming procedures. In principle, in vivo labeling is a simple and easy method but has a problem of off-target labeling of undesired amino acids, which is called isotope scrambling or dilution. Isotope scrambling is usually tolerable for amino acids Ala, Arg, Asn, Cys, His, Ile, Lys, Met, Pro, and Trp, which are end products in the metabolic pathways, whereas Asp, Glu, and Gln, which occupy an earliest or intermediate position in a metabolic pathway, are highly prone to isotope scrambling. The remaining amino acids (Gly, Phe, Leu, ser, Thr, Tyr and Val) show a weak-to-medium tendency for isotope scrambling. The labeling of undesired amino acids can be minimized by means of auxotrophic cells that lack the synthesis enzyme producing a specific amino acid, or by incorporating specific inhibitors of a certain amino acid synthesis enzyme into the biosynthetic pathway. Recently, advanced methods for efficient and simple isotope labeling were also developed using common prototrophic *E. coli* strains [47,48]. Thus, the amino-acid selective labeling combined with modern NMR techniques is frequently employed as a useful tool for resolving resonance assignments. Nevertheless, this approach is not a panacea because of persistent overlaps and ambiguous connectivity data.

### 2.2. The Trends and Choice of Membrane-Mimetic Media for Structural Studies 

**Figure 2:**
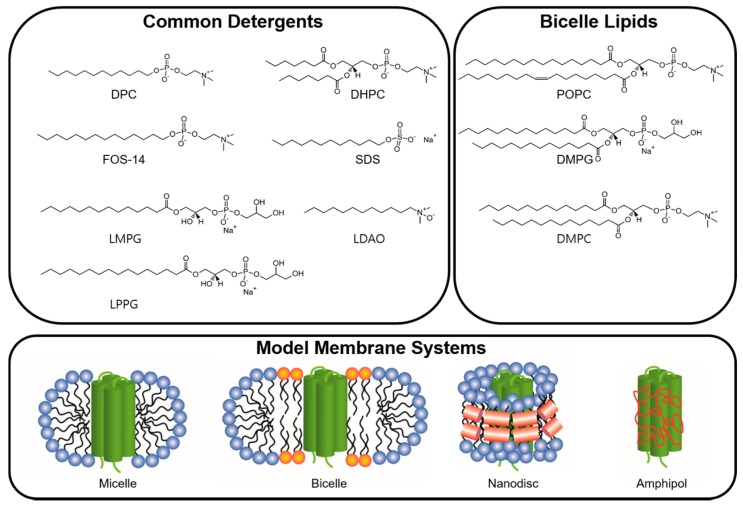
Common detergents and lipids used to prepare membrane environment for solution NMR studies on α helical transmembrane proteins. Several common detergents for forming micelle and lipids for forming bicelle are shown. Figures shown in the bottom are schematics of the morphologies for the micelle, bicelle, nanodisc and amphipol.

In the cell biological membranes play a vital role as semipermeable barriers composed of complex assemblies of lipids and proteins. Cellular membranes are bilayer constructions in which the polar head groups are exposed to an aqueous environment and the hydrophobic tail groups are packed behind the hydrophilic head groups in accordance with the architecture of the majority of lipid membranes, where a lipid contains two hydrophobic hydrocarbon tails connected to a polar head group. Hydrophobic residues in integral MPs are frequently exposed to the outside, in contrast to soluble proteins, thus allowing MPs to be held in the membrane by hydrophobic interactions between the hydrocarbon chains of the lipids and exposed hydrophobic regions of the proteins. A Native membrane environment is usually not available for in vitro structural studies on integral MPs; instead, these studies are conducted on isolated proteins kept in artificial medium, containing e.g., detergents, bicelles, nanodiscs and amphipols (Figure 2).

#### 2.2.1. Detergents

A detergent is the most common membrane-mimetic medium for structural studies on MPs by solution NMR. A detergent molecule consists of a polar head group at one end and a long hydrophobic carbon chain at the other end. The polar head groups are facing the aqueous environment via hydrogen bonding with water molecules, while hydrophobic regions aggregate via hydrophobic interactions [49]. In particular, when detergents are introduced into an aqueous solution, the existing hydrogen-bonding network supported by water molecules is disrupted, and water molecules are rearranged around the nonpolar region of the detergent thus forming hydrogen bonds. As detergents are added to the solution, its molecules self-associate for reducing the total water-accessible surface of the nonpolar region of the detergent complex; this phenomenon is driven by the favorable thermodynamic effect on the bulk water phase [50] and is called micellization. Each detergent molecule is in the form of a monomer below a certain concentration, but above that concentration, called *critical micelle concentration* (CMC), detergent molecules associate to form stable and non-covalent aggregates known as micelle [51]. Another important parameter of micelles is the aggregation number, which means the number of detergent monomers present within a single micelle [52]. Ideally, with the knowledge about CMC and aggregation number of detergents, several important parameters can be calculated using Equations (1)–(3):

Total detergent concentration = [CMC] + [free micellar detergent] + [protein-associated detergent](1)

Micelle concentration = ([total detergent concentration] − [CMC])/aggregation number(2)

Micelle aggregate molecular weight = aggregation number × monomer molecular weight(3)

Micelle size is dependent on the balance of “opposing force”, which are an energetically unfavorable force due to close proximity of head groups and a favorable force between hydrophobic tails in the micelle [50]. The important determinant of micelle shape is the maximal possible extension of the hydrocarbon chain [53]. For example, the shapes of dodecylphosphocholine (DPC), *n*-decyl-β-D-maltoside (DM), dihexanoylphosphatidylcholine (DHPC) and Lyso-myristoyl phosphatidyl-glycerol (LPPG), which are commonly used in NMR studies are prolate, oblate, prolate and oblate, respectively [53]. In contrast, when a MP integrated into micelle, the aggregate size and shape are not always relevant to the size and shape of the corresponding protein-free micelles [54].

**Figure 3:**
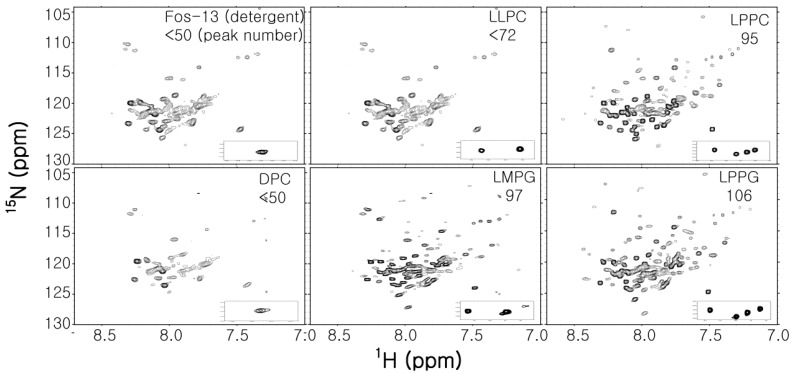
Comparison of the ^1^H-^15^N TROSY spectra of full-length WT Cav3 in various detergent micelles. Detergent name and the number of observed peaks are presented in each spectrum. Caveolin3 is an unusual shaped integral MP composed of soluble N-terminal region (54 residues), helix-break-helix integral membrane region (44 residues), which does not penetrate the cellular membrane and C-terminal region located on the membrane surface (53 residues) [60]. The expected number of backbone amides is 153. All spectra were recorded at 318 K on a Bruker 800 Mhz NMR machine.

Detergent molecules can be classified into three types; ionic detergents, non-ionic detergents and zwitterionic detergents. Ionic detergents can be subdivided into anionic and cationic ones, according to their net charge. Such detergents are useful for disrupting protein-protein interactions and their CMC is affected by ionic strength and is relatively unaffected by temperature changes. Non-ionic detergents consist of an uncharged hydrophilic head group and are suitable for breaking lipid-lipid and lipid-protein interactions. These detergents can gently solubilize MPs without disrupting the native structure of proteins; therefore, MPs inserted into an expression cell membrane are usually extracted using nonionic detergents such as Triton^®^-x-100, DM and *n*-dodecyl-β-D-maltoside (DDM). Their CMC is affected by temperature changes and relatively unaffected by ionic strength. Zwitterionic detergents such as DPC, 3-[(3-Cholamidopropyl)dimethylammonio]-1-propanesulfonate (CHAPS) and Zwittergents have a combination of properties of ionic and non-ionic detergents and are suitable for disruption of protein-protein interactions. Some properties of detergents commonly used for structural studies by solution NMR are listed in Table 1. Indeed, NMR signals in the spectrum of integral MPs can be completely changed by the use of a different kind of detergent for solubilization (Figure 3).

**Figure 4:**
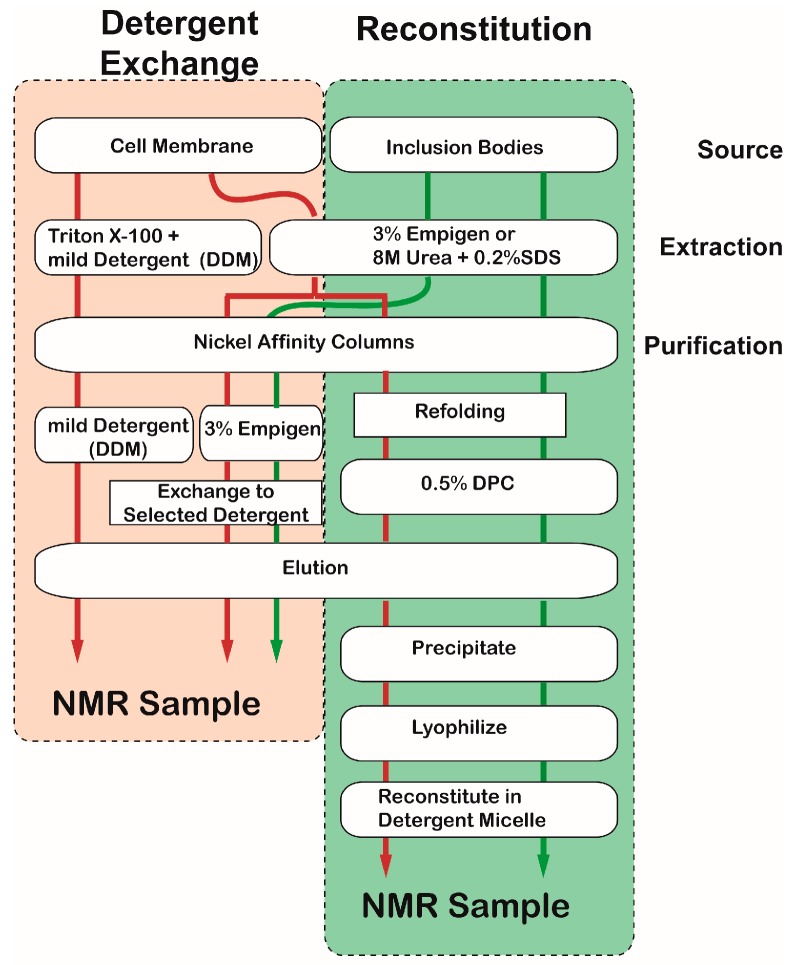
Flow chart representing two simple methods to prepare NMR samples incorporating with desired detergent. The red and green arrows represent the purification pathways using cell membrane fraction and inclusion bodies, respectively.

Two simple methods can be applied to incorporate an MP into detergent micelles: “detergent exchange” and” reconstitution” (Figure 4). The brief overview of the two sample preparation protocols is presented in Figure 4. The structure of an MP in the membrane fraction may be close to the native structure; thus, the protein can be extracted with a mild detergent such as Triton^®^-x-100. Proteins in inclusion bodies can be extracted using a harsh detergent or a combination of urea and SDS. Fully or partially denatured MPs are refolded while being incorporated into a desired detergent by means of a nickel column. Both methods can produce high-quality samples, but the resulting NMR spectra sometimes vary [61]. Perhaps, this variability is caused by differences in conformation or dynamic states of the MP among different samples. At this moment, it is difficult to say which method is better.

Besides the use of single detergents for structural studies, mixed micelles can also serve as membrane-mimetic media. The advantage of mixed micelles is the possibility to adjust the basic features and thickness of the micelles. The characteristic micelle thickness depends linearly on the detergent mixing ratio; this situation implies a straightforward method to engineering of micelles of particular thickness by mixing detergents. Lesley and colleagues reported that NMR spectra of integral MPs can be improved by matching of micelle dimensions (controlled by mixing detergent ratio) to the protein’s hydrophobic surface [62]. In this work, ^1^H-^15^N TROSY spectra of an MP called TM0026, which is a small protein (68 residues) containing two transmembrane α helices, in different detergents such as DM, DDM, *n*-decylphosphocholine (FC-10), DPC, DDM/FC-10 mixture and LPPG/DHPC mixture were compared and showed interesting results. Poor NMR spectra were observed in the samples containing DDM or FC-10, which form either smaller and thinner or larger and thicker micelles, respectively, as compared to DM and DPC. The DDM/FC-10 and LPPG/DHPC mixtures—where DDM/DPC served as an extension of the four detergents under study and the LPPG/DHPC was chosen because TM0026 in each pure micelle solution was either insoluble (DHPC) or yielded a low-quality NMR spectrum (LPPG)—formed micelles of the size similar to that of DM and DPC micelles and consequently provided good NMR spectra. These results suggest that a proper mixture of micelles with a limited set of detergents may be rationally designed for NMR structural studies.

#### 2.2.2. Non-Micellar Media

Detergent micelles are most frequently used for structural studies on MPs. Nevertheless, detergents often cause problems by affecting protein function and stability while creating a membrane environment [63] because micelles generally possess highly dynamic properties that frequently lead to protein unfolding and aggregation. In this regard, multiple-span MPs containing a large hydrophobic moiety are more likely to be exposed to an aqueous environment at an inopportune moment. Moreover, another drawback of detergents is that they can disrupt protein-protein or protein-lipid interactions, which are closely related to protein function. For that reasons, alternative membrane-mimetic media are desirable for structural studies on α-helical transmembrane proteins. Among the non-micellar media, isotropic bicelles, nanometric lipid bilayers and amphipols represent most promising alternatives.

A bicelle represents an intermediate structure between a vesicle and traditional micelle. An ideal bicelle is composed of a mixture of the central flat part formed by lipids and the edge of a disc-shaped assembly formed by detergents as shown in Figure 2. Bicelles usually provide a more native membrane environment for structural studies in comparison with micelle. So far, a number of bicelle systems have been developed and characterized. Among them, a mixture of the detergent DHPC and a lipid, dimyristoylphosphatidylcholine (DMPC) or 1-palmitoyl-2-oleoyl-sn-glycero-3-phospho-choline (POPC) or 1,2-dimyristoyl-sn-glycero-3-phosphoglycerol (DMPG), is a representative product, which has been used for successful determination of tertiary structure of α helical MPs [64,65,66]. Sanders and colleagues conducted structural characterization of C99, the 99-residue transmembrane C-terminal domain of amyloid precursor protein, in various DHPC-based bicelles [67]. This work indicated that the overall structure of C99 underwent no appreciable changes, but modest membrane topological adjustments were observed when C99 was reconstituted in various bicelles. Thus, to study an interaction e.g., taking place on the membrane surface, the influence of bicelle composition should be considered. Meanwhile, bicelle composition can be described by the parameter q, a molar ratio between the lipid and the detergent above CMC, which is a critical factor for understanding bicelle morphology. The parameter q can be calculated using Equation (4):

q = total molarity of lipid/(total molarity of detergent − CMC of detergent)(4)

For example, the q values of DHPC-DMPC bicelles, a frequently used medium for structural studies by solution NMR, are typically in the range of 0.25 to 0.5, which indicates that this bicelles are lipid-poor and detergent-rich. As with micelles, bicelles also have a disadvantage: a small amount of the detergent can diffuse into the protein and make it unstable. Moreover, the q value has to be maintained to avoid any phase transition; however, it is not easy to keep q value constant during some experiments involving a buffer exchange or temperature change.

Nanometric lipid bilayers, often refered to as nanodiscs, have been designed to creat a nativelike lipid bilayer without using detergents. A nanodisc is composed of a noncovalent assembly of a phospholipids bilayer in a liquid crystal states, surrounded by a dimer of an engineered lipoprotein called membrane scaffold protein (MSP) [68,69]. In vitro reconstitution of nanodiscs allows for creation of a nearly native membrane environment (from desired lipids), which supports protein stability as well as its functionality. An MP incorporated into a nanodiscs is highly stable and allows for recording of NMR spectra at high temperature for hours and even days. Indeed, high-quality NMR spectra can be obtained [70,71]. Furthermore, Lee and colleague successfully measured residual dipolar coupling (RDC) values of a β-barrel outer MP OmpX in nanodiscs and refined the structure [72]. Another advantage of nanodisc is the possibility to adjust the size and shape via MSP engineering [73]. 

Amphipols are amphipathic polymers, consisting of both numerous hydrophilic and abundant hydrophobic groups and have been proven to be a valid alternative to detergents [74]. This compound gets attached at multiple point along the transmembrane region of an MP, which can thus stay water-soluble in its native conformation regardless of its types and size. Amphipols cannot extract MPs from biological membranes because they are usually ineffective as dissociating surfactants [75,76]. Usually, MPs are first extracted and purified using a detergent, and then an amphipol is added to form an MP-detergent-amphipol complex, which can include lipids of interest as an option. Next, the detergent can be removed in two simple way; dilution under its CMC or by means of bio-beads absorbing detergent [77]. Many amphipols have been developed, but among them, the most promising are A8-35, which is the best characterized and compatible with NMR studies because deuterated and perdeuterated forms are available, and sulfonated amphipols (SAPols) and glucose-based, non-ionic amphipols (NAPols), both are insensitive to acidic pH but not commercially available yet [78]. Even though a structure of an MP combined with an amphipol has not been determined yet, many studies revealed that an MP-amphipol complex can yield high-quality NMR spectra [78,79]. One possible drawback of amphipols is that their morphology is actually quite different from that of a lipid bilayer.

#### 2.2.3. Analysis of Successful Membrane Mimetics 

**Figure 5:**
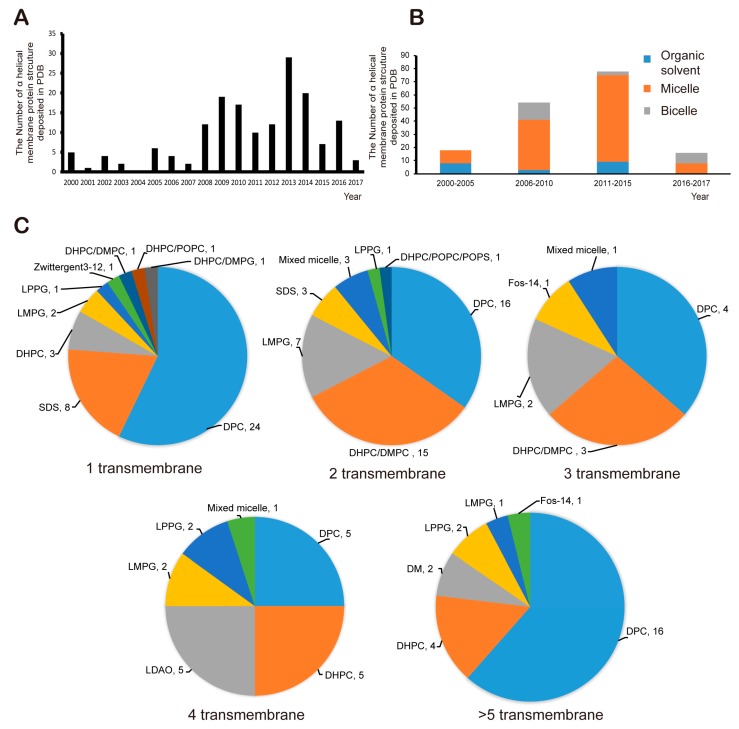
The analysis of successful structure determinations of α helical transmembrane proteins. (**A**) the number of success stories by year (**B**) the kind of membrane model system used in success stories by year (**C**) the kind of membrane mimetic components depending on the number of membrane spanning helices (component, successes number), and organic solvents as membrane environments were excluded from the analysis.

As a result of various efforts, 166 structures of α-helical transmembrane proteins determined by solution NMR have been deposited on the PDB website since 2000 (structures deposited before 2000 were excluded from the analysis in the sector, Figure 5A). Since the mid-2000s, more than 10 structures have been deposited annually, but the total number of successfully determined structures of α-helical MPs is still small. In other words, research into MP structure using solution NMR is still a challenge. One of the major bottlenecks is that it is extremely difficult to obtain high-quality NMR spectra. There are many possible reasons for this problem, but for the most part, membrane mimetics for stabilization of MPs are not suitable for this purpose. Usually, proper membrane mimetics are selected by a trial-and-error approach based on the MP sample at hand; this process is time-consuming and expensive. Nonetheless, successful cases have given rise to minimal guidelines for selecting artificial membrane. Every analysis deals with α-helical integral MPs in this section of the article. Until the mid-2000s, micelles and organic solvents such as chloroform, methanol, and dimethyl sulfoxide (DMSO) had been frequently used as shown in Figure 5B. Since the mid-2000s, successful use of bicelles has been demonstrated, and the number of success stories involving micelles has increased. If we look more closely at the use of micelles, among the 124 success stories, DPC was used in more than a half of the cases, followed by LMPG, DHPC, SDS and mixed micelles (Figure 5C). In case of bicelles, the DHPC-DMPC mixture was used in 19 cases out of 22 success stories. As shown in Figure 5C, for single-span MPs, DPC has been used in the vast majority of the 42 success stories, followed by SDS, DHPC and LMPG. As for two-span MPs including heterodimeric complexes inside a membrane environment, DPC and DHPC-DMPC bicelle systems have similar success rate at 16 and 15 cases, respectively, followed by LPPG, mixed micelles and SDS. Bicelles have not been used for a ≥ 4-span MP, whereas DPC is suitable for any type integral MPs. Overall, the reason why DPC and DHPC/DMPC bicelle are frequently used, of course, is that a protein is well folded and stabilized in that environment, but perhaps, whether it is possible to obtain a commercially deuterated membrane-mimetic component is also important because, as mentioned above, to obtain NOEs from a protein side chain, other components generally should be deuterated.

In short, there are two main membrane mimetic systems for solution NMR studies: micelle and non-micellar system. Micelle is a fast equilibrium system between free single detergents and micellar detergents that can be used extensively in any membrane protein form. In practice, micelles often give high quality NMR spectra. It is a good idea to start NMR studies of membrane protein with selected detergent such as DPC and DHPC for the first time. Nevertheless, bicelle and nanodisc sometimes give the better NMR spectra because these are more similar to real bilayer cell membrane environment. To investigate the interaction occurred inside membrane, in particular, bicelle membrane mimetic should be the first consideration. On the other hand, since nanodisc does not use detergent, a kind of denaturant, it can be an ideal membrane environment system to study on the loop connecting membrane spanning helices or protein with loss of function in the other membrane mimetic media. Recently, Wagner and colleagues developed the uniformed nanodiscs of various sizes from ~8.5 nm to ~180 nm, and they demonstrated the superiority of these nanodiscs by showing high quality NMR spectra [80]. Until now, DPC micelles have been the most successful membrane mimetic, but nanodiscs are expected to be actively used in the future.

## 3. Illustration of Successful Structure Determination of Integral MPs 

### 3.1. Structure of the Mitochondrial Translocator Protein in Complex with a Diagnostic Ligand

**Figure 6:**
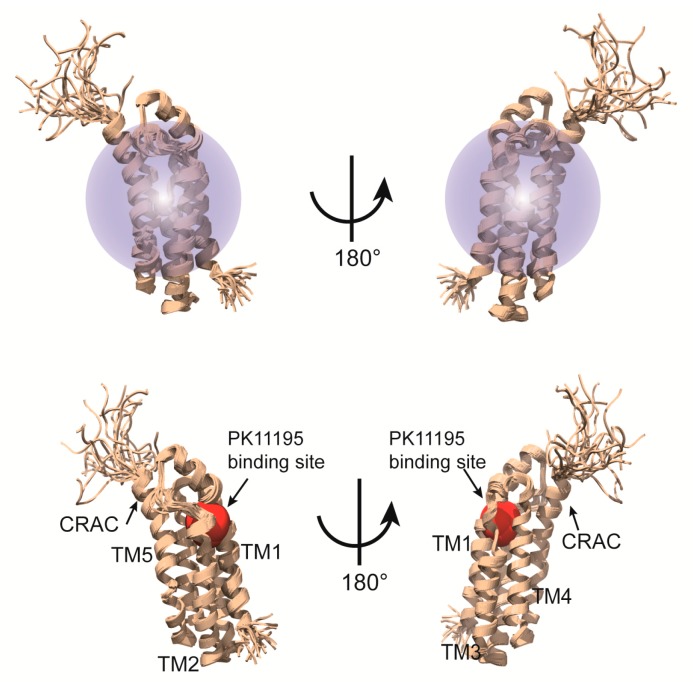
High-resolution solution NMR structure of the mTSPO-PK11195 complex [81]. Upper panel represents TSPO-11195 complex incorporated into DPC micelle. Lower panel shows 5 membrane spanning helices, PK11195 binding site and CRAC motif.

The structural studies on a complex of a transmembrane protein with its ligand can provide key insights into the molecular mechanism of action. On the other hand, ligand binding is frequently sensitive to membrane-mimetic media and usually NMR signals from the ligand overlap with those from the detergent; therefore, structural analysis of a ligand-binding integral MP is challenging. Zweckstetter and colleagues determined the structure of mitochondrial translocator protein in complex with its high-affinity ligand [81]. The translocator protein (TSPO) is mainly expressed in mitochondrial membranes of steroidogenic tissues [82,83]. TSPO is known as a peripheral benzodiazepine receptor and a transporter of cholesterol from the cytosol into mitochondria [82,84,85]. This protein also performs important functions in apoptosis and stress adaptation [86,87]. The diagnostic radio ligand, [^11^C]1-(2-chlorophenyl)-*N*-methyl-*N*-(1-methylpropyl)-3-isoquinoline-carboxamide (PK11195), which can be detected and visualized by the position emission tomography (PET), binds to TSPO with nanomolar affinity and serves as a biomarker of inflammation in the brain [88]. Based on sequence analysis, 18-kDa TSPO was predicted to have five membrane-spanning helices. In this work, mouse TSPO was overexpressed in *E. coli* and the purified protein was reconstituted in DPC micelles. TSPO as is in DPC micelles provided highly overlapping and clustered NMR signals in the middle region because TSPO may have dynamic properties in that environment. In contrast, ligand binding led to dramatically improved NMR spectra owing to stabilization of the protein. Backbone resonance peaks were assigned with the help of TROSY-based typical 3D spectra using a ^15^N/^13^C/^2^H-labeled TSPO-(R)-PK11195 complex and 2D spectra as well as 3D ^13^C-edited NOESY-HSQC (mixing time of 200 ms) and ^15^N-edited NOESY-HSQC (mixing time of 200 ms) spectra in a complex sample in which selected amino acids (Ile, Lys, Gly, Pro, Trp and Arg) were ^15^N/^13^C-labeled while the other residues were perdeuterated, with natural abundance of carbon and nitrogen isotopes. A total of 3362 NOEs including over 1500 NOEs of a medium and long range and 61 NOEs between protein and ligand were acquired and helped to determine high-quality tertiary structure of the TSPO-PK11195 complex. Deuterated DPC was used to avoid signal interference between DPC and protein sidechains. The structure of the complex was calculated using CYANA 3.0 [89] and refinement was carried out in Xplor-NIH 2.33 [90] without any other aids such as RDC or paramagnetic relaxation enhancement (PRE). As a results of their efforts, those researchers could reveal the complex structure at atomic resolution and learned how TSPO contacts PK11195 (Figure 6). As mentioned above, MPs are major drug target in the pharmaceutical industry, and therefore the structural information on MP-drug interactions can be used for rational drug design. In many cases, the binding site in a protein for a ligand can be traced using chemical shift titration, which is useful even for weak binding. Nevertheless, this method is inaccurate under certain circumstances, for example, when ligand binding induces conformation changes in the protein. As an alternative method, acquisition of NOEs between the protein and ligand is a more accurate approach. Nonetheless, if the residues located in the loop connecting membrane spanning helices are involved in the ligand binding, the results analysis is possibly more complicate because these residues in the loop is more mobile than those in the helices, which can cause the conformational heterogeneity [91,92]. In the above-mentioned project, chemical shift titration was not effective at identifying the binding site because the protein underwent global conformation changes upon binding of the ligand, PK11195. Instead, those researchers could successfully obtain NOEs directly indicating binding residues of the protein toward the ligand. The determined structure of the complex also revealed that the CRAC motif, essential for cholesterol binding [93,94], is distinct from the ligand-binding site.

### 3.2. Unusual Architecture of the p7 Channel from Hepatitis C Virus

One recent successful application of micelle in solution NMR is to reveal the structural organization and mechanism of p7 cation channel from hepatitis C virus. This cation channel is formed by oligomerization of p7, a small membrane protein, and is known to be inhibited by amantadine and rimantadine, the drug to treat influenza infection by block influenza M2 proton channel. Ouyang et al. find that the p7 in dodecylphospocholine (DPC) micelle forms a hexamer by using negative stain electron microscopy, in consistent with previous EM studies. NMR and ITC binding studies further show that rimantadine binds specifically to p7 in DPC micelles, indicating the hexamer is functional.

To understand the structural mechanism for the p7 cation channel, Ouyang et al. has solved its NMR structure in DPC micelle, which is challenging as the size of protein in micelle is ~60 kDa with protein itself 40 kDa and DPC micelle ~20 kDa. The following protocols were used to successfully get the structure [18].

First, the backbone chemical shift assignment was obtained through TROSY-based sequential assignment experiment: HNCA, HN(CO)CA, HNCACB, HN(CO)CACB, HNCO and HN(CA)CO. the sidechain chemical shift and NOE assignment was obtained through ^15^N-edited NOESY-TROSY-HSQC and ^13^C-edited NOESY-HSQC experiment. The backbone ^1^H-^15^N residual dipolar coupling was measured using gel aligned NMR sample. The Xplor-NIH program was used to calculate the monomer structure by using backbone dihedral angle restraint derived from backbone chemical shift, the local NOE restraint and the RDC restraint. The structure ensemble obtained in this step has a backbone RMSD of 0.74 Å of the structural region.

**Figure 7:**
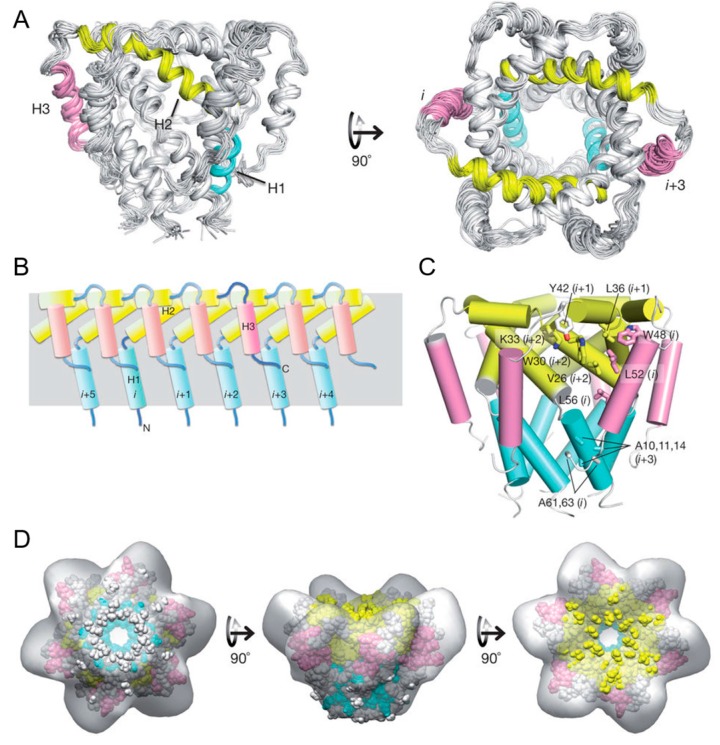
Solution NMR structures of p7 hexamer (**A**) ensemble of 15 low-energy structures cartoon represents (**B**) the intermonomer interactions among the H1 (blue), H2 (yellow) and H3 (pink) helical segments in the hexameric assembly and (**C**) the global arrangement of helical segments and amino acids (**D**) Fitting the solution NMR structure to the 16 Å EM map (EM database ID 1661) Reprinted by permission from Macmillan Publishers Ltd: Nature (18), copyright (2017).

Second, the intermonomeric NOE was measured by 15N-NOESY-TROSY-HSQC on a special NMR sample that has equal amount of ^15^N/^2^H- and ^13^C/^1^H-labeled monomer. Each intermonomeric NOE contribute to 6 equal distance restraint for the complex structure calculation to satisfy the hexamer symmetry. Xplor-NIH was used first to assemble the complex with intermonomeric NOE, then further refine the structure with RDC restraint, and finally use all restraint to get the final structure ensemble. As shown in the Figure 7, the NMR structure of the hexamer shows a funnel-like structure, with the ith monomer p7 also interacts with i + 2, i + 3 besides its neighbor monomer i ± 1. The high quality structure also suggests that there exist three different filter: the first one is composed of conserved Arg/Lys 35 that forms a positive guanidinium ring in hexamer to filter out all anions; the second is composed of conserved ASN/His 9 that form a polar ring in hexamer to dehydrate cation ions; The third is ILE 6 forms a hydrophobic ring to serve as hydrophobic filter to prevent water trafficking. 

To reveal the inhibition mechanism of amantadine on p7 channel, the complex structure of p7 channel and amantadine was calculated by the distance restraint derived from protein-ligand NOE. This structure, although not taking into consideration any long range effects of the inhibitor on protein structure, shows that the hydrophobic binding pockets is located between pore-forming and peripheral helices. This binding site was further confirmed by mutation studies, that mutation in the region significantly reduces the channel activity. As the flexibility in this region is important for channel opening and closing, the inhibition might be caused by that inhibitor binding rigidify this region. Functional and other biophysical studies further support these conclusions.

### 3.3. Implication of the Differing Roles of the β1 and β3 Tramsmembrane and Cytoplasmic Domains 

**Figure 8:**
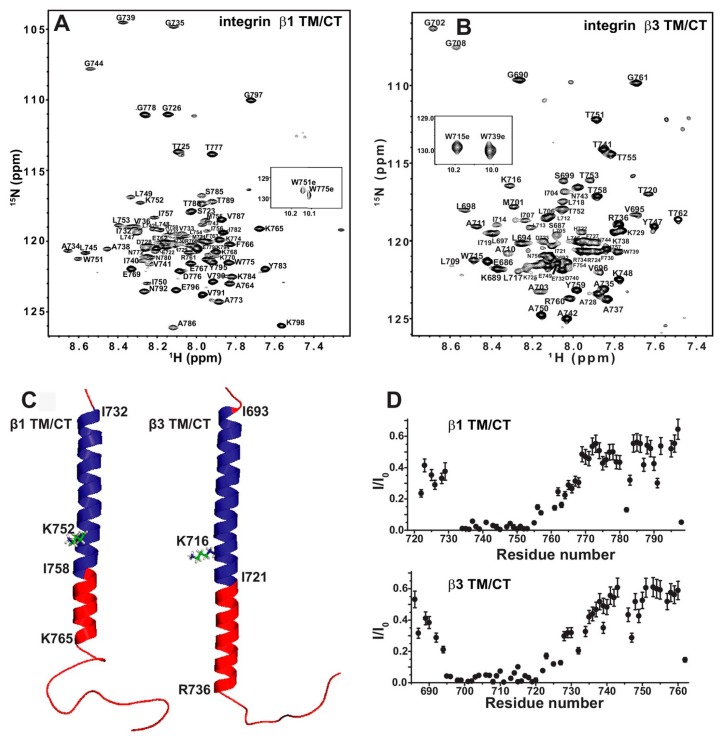
The β1 and β3 transmembrane and cytosolic domains have distinct structures. (**A**) 800 MHz ^1^H-^15^N TROSY spectrum of the WT integrin β1 TM/CT with peak assignments shown; (**B**) 900 MHz ^1^H-^15^N TROSY spectrum of the WT integrin β3 TM/CT with peak assignments shown; (**C**) Structural comparison of integrin β1 and β3 TM/CT in DHPC/DMPC bicelles; (**D**) Assessment of exchange of protons between backbone amide sites and water. Reprinted from Lu et al. [95].

Sanders and colleagues have used bicelles as membrane mimetic systems to study the structure and interactions of integrin transmembrane/cytoplasmic tail (TM/CT) domain [95]. Integrin is type I transmembrane protein that mediates the interaction between cell and extracellular matrix, thus plays important roles in cell functions. There are 24 heterodimeric integrins formed specifically by 18α and 8β subunit. Many functional studies have shown that TM/CT domain is key to integrin activation. Studies on platelet integrin αIIb/β3 TM domain has shed light on the activation mechanism. However, as β3 integrin family only has two members, whether such mechanism can be applied to other integrins is not clear. Lu et al. studied the TM/CT domain of three β1 integrins: α1β1, α2β1 and α5β1 and one β3 intetrin αIIbβ3. The initial bicelle screening show the DMPC/D6PC bicelle give the best NMR spectra and set as the memberane mimetic system for further NMR structural studies. They have obtained the backbone chemical shift assignment of both integrin β1 and β3 TM/CT (Figure 8A,B) by using TROSY-based experiment HNCACB, HN(CO)CACB, HNCA, HN(CO)CA, HNCO, HN(CA)CO in combination with ^15^N-TROSY-HSQC of ^15^N-residual selective labeling and ^14^N-residual reverse labeling. As shown in the Figure 8C, the secondary structures obtained from chemical shift data show that the TM/CT domain of integrin beta3 has a much longer α helix than that of integrin β1, which is further supported by the exchange profile between protein amide proton and water (Figure 8D). The binding of α/β TM/CTs was studied by NMR titration with one subunit is ^15^N-labeled and the other is unlabeled. As the previous NMR studies of αIIbβ3 TM/CTs show that negative charged lipids (POPS) can enhance the αIIbβ3 TM binding, the binding of different integrin TM/CT was carried out in POPC/POPS/DHPC bicelles. The measured dissociation constant shows that α5/β1 has similar binding affinity to αIIbβ3 (0.17 mol % vs. 0.15 mol %), the α2/b1 and α1/β1 have much weaker binding (>2.5 mol %). this clearly show that the binding affinity varies a lot for different integrin, even for the same β1 subunit. Previous studies of αIIbβ3 show that the conserved snorkeling lysine, located at the interface between membrane and aquatic phase, is key to the αIIbβ3 TM complex formation and thus to the activation. Mutation of this lysine to glutamate in β3 leads to a hydrophobic patch of 5 residues after this residue move out of membrane as shown by paramagnetic probe induced NMR peak intensity reduce/disappear, the complex dissociation as observed by NMR titration, and integrin activation as shown in in vivo functional studies. Further studies show that even though the lysine mutation has similar effects on β1 and β3 TM/CT topology, it does not change the membrane topology in either case. However, it has significant different effects on binding affinity on different integrins, and has opposite effects on β1 and β3 integrin function. The NMR titration studies of integrin β1/3 binding to its activator protein talin show that the lysine mutation significantly reduces β3/talin binding, not β1/talin binding. Thus, the mechanism derived from platelet integrin cannot be assumed to be true for all other integrins and further studies are necessary to reveal how different integrins function differently.

### 3.4. Architecture of the Mitochondrial Calcium Uniporter

Studies on the membrane-associated region by solution NMR alone are still very challenging, therefore, a combination of more than two methods for structural studies is effective nowadays. Recently, Chou and colleagues reported the pentameric structure of a protein called mitochondrial calcium uniporter (MCU), including the transmembrane region solved using a combination of solution NMR and EM [10]. MCU is regarded as a pore unit of a uniporter holocomplex, composed of MCU’s inactive paralog MCUb, an accessary single-pass transmembrane peptide called EMRE, and EF-hand Ca^2+^-binding proteins MICU1 and MICU2 [96]. MCU was predicted to be at least a tetramer; therefore, the size of this full-length protein complex is likely to be over 160 kDa, which is an insuperable barrier to structural analysis by the current solution NMR technology. For overcome this problem, they used two approaches: deletion of *N*-terminus and a combination of solution NMR and EM. Because the N-terminal domain (165 residues) is believed to not be involved in channel activities, they screened various N-terminal domain-deleted constructs and then selected one from *C. elegans* (cMCU-ΔNTD), which can be overexpressed in *E. coli* expression system. Structural studies on big proteins using solution NMR have generally involved the “divide and conquer” approach. In this work, Fos-14 was used for extracting and stabilizing cMCU-ΔNTD, and all experiments were conducted in the presence of same detergent.

**Figure 9:**
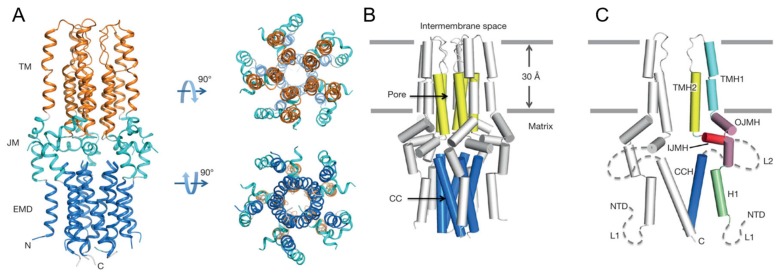
High-resolution solution NMR structure of the cMCU-ΔNTD. (**A**) Structures are displaying three distinct layers; the transmembrane (TM; orange), juxtamembrane (JM; cyan), and extramembrane domain (EMD; blue) regions, respectively; (**B**) Cartoon representation of the cMCU-ΔNTD pentamer showing the formation of the uniporter core (yellow) and the coiled-coil pentamer formed by CCH (marine); (**C**) Cartoon representation showing the folding of individual subunits. Reprinted by permission from Macmillan Publishers Ltd.: Nature (10), copyright (2017)

This protein was revealed to be a pentameric complex, with the size reaching 90,375 Da. As mentioned above, they performed negative-stain EM analysis to facilitate structure determination by NMR and obtained a reconstructed 3D density map refined to a resolution of ~18 Å, where a roughly cylindrical shape with five-fold symmetry was observed. Next they proceeded to NMR experiments to solve the structure at high resolution at an atomic level. Local structures of monomers were determined mainly using local distance restraints derived from NOEs and backbone dihedral restraints derived from a chemical shift, after which assembly of the oligomer with intermonomer distance restraints was performed [8,18,97]. To obtain intermonomer distance restraints, a 1:1 mixed differentially labeled sample, such as ^15^N/^2^H-labelled cMCU-ΔNTD and (15%, ^13^C)-labelled cMCU-ΔNTD, was used to measure NOEs between the ^15^N-H of one subunit and non-exchangeable aliphatic protons of adjacent monomers. In this experiment, the detergent had to be deuterated so as not to interfere with the NOE examination. In other words, the choice of membrane-mimetic media for this kind of experiments has a limitation in that the deuterated medium usually has to be available commercially. Finally, 2070 local and 150 long-range intramonomer distance restraints and 220 intermonomer restraints were collected using ^15^N-edited NOESY-TROSY and ^13^C-edited NOESY-HSQC pulse programs. The structure ensembles were calculated using the Xplor-NIH software [90]. Of note, this structure was calculated without the help of restraints derived from RDC and paramagnetic relaxation enhancement (PRE), which are commonly used for structure calculation of MPs. Nevertheless, those researchers could obtain a high-quality solution structure whose Ramachandran plot statics (calculated in PROCHECK (European Bioinformatics Institute, Cambridge, UK) were as follows: most favored (86.3%), additionally allowed (11.0%), generously allowed (1.8%) and disallowed (1.0%). As shown in Figure 9, the resulting structure of cMCU-ΔNTD represents a well-packed pentamer in which the inner core of the complex is formed by the transmembrane helix (residues 244–260) and a coiled-coil helix (residues 293–316). Their structure revealed that DXXE looks like the mouth of the pore motif that is structurally critical for Ca^2+^ flow, and the calculated structure in this work turned out to be a closed conformation of the channel, in line with another report [98,99].

## 4. Conclusions

Structural studies on MPs including G protein-coupled receptors and ion channels, are an important research area for understanding various biological phenomena and are of particular interest to pharmaceutical companies because these proteins are major targets for drug development. Nonetheless, structural analysis of many MPs has yet to be achieved because of the various difficulties. When solution NMR is employed to study MP structures, acquisition of a sufficient amount of MP, a proper membrane mimetic medium, and high quality NMR spectra are needed. In this review, general approaches to preparation of NMR samples such as protein-labeling techniques as well as the promising artificial membrane such as micelle, bicelle, nanodisc, and amphipol were described. The analysis of membrane-mimetic media based on successful cases of structural determination by solution NMR are expected to facilitate the selection of such media for structural analysis and characterization of MPs. The four successful example above represent structural studies on a complex of an MP with its ligand, an MP with an unusual shape, heterodimeric MPs, and analysis of an MP using a combination of solution NMR and EM, respectively. These success stories are expected to serve as guidelines for structural studies of other α-helical integral MPs.

## Figures and Tables

**Table 1:** Properties of commonly used detergents for structural studies.

Detergent	Charge	Molecular Weight	Critical Micelle Concentration (mM)	Aggregation Number	Reference
Dodecylphosphocholine (DPC)	Zwitterionic	352	1.5	50–60	[54]
Lyso-myristoyl phosphatidylglycerol (LMPG)	Anionic	478	0.2–3	ND	[55]
Dihexanoylphosphatidylcholine (DHPC)	Zwitterionic	454	15	19–35	[56,57]
Sodium dodecylsulfate (SDS)	Anionic	288	1–7	62–101	[54]
Lyso-palmitoyl phosphatidylglycerol (LPPG)	Anionic	506	0.02–0.6	125	[56]
*N*-Lauroyldimethyl amineoxide (LDAO)	Zwitterionic	229	2	69–73	[54]
β-Dodecylmaltoside (DDM)	Nonionic	511	0.2	110-140	[54]
*n*-Tetradecylphosphocholine (Fos-14)	Zwitterionic	379.5	1.2	108	[58]
Zwittergent 3–12	Zwitterionic	335.6	3	55–87	[59]
